# Conditional survival and hazards of death for peripheral T-cell lymphomas

**DOI:** 10.18632/aging.202782

**Published:** 2021-03-26

**Authors:** Hongye Gao, Xinqiang Ji, Xin Liu, Lan Mi, Weiping Liu, Xiaopei Wang, Jun Zhu, Yuqin Song

**Affiliations:** 1 Key Laboratory of Carcinogenesis and Translational Research (Ministry of Education), Department of Lymphoma, Peking University Cancer Hospital and Institute, Haidian 100142, Beijing, China; 2 Key Laboratory of Carcinogenesis and Translational Research (Ministry of Education), Department of Medical Record Statistics, Peking University Cancer Hospital and Institute, Haidian 100142, Beijing, China; 3 State Key Laboratory of Molecular Oncology and Department of Radiation Oncology, National Cancer Center, Cancer Hospital, Chinese Academy of Medical Sciences (CAMS) and Peking Union Medical College (PUMC), Chaoyang 100021, Beijing, China

**Keywords:** lymphoma, T-cell, peripheral, survival analysis, prognosis

## Abstract

Typically, peripheral T-cell lymphoma (PTCLs) prognosis is estimated using overall survival before treatment. However, these estimates cannot show how prognosis evolves with the changing hazard rate over time. Patients (n = 650) with newly diagnosed PTCLs were enrolled retrospectively. After a median follow-up of 5.4 years, angioimmunoblastic T-cell lymphoma, peripheral T-cell lymphoma, not otherwise specified (PTCL, NOS) and NK/T cell lymphoma had initially lower 3-year conditional overall survival (COS3; i.e., the 3-year conditional overall survival was defined as the probability of surviving an additional 3 years) and higher hazards of death (26–44.3%). However, after 2 years, the COS3 increased and the death risk decreased over time, whereas anaplastic lymphoma kinase-positive anaplastic large-cell lymphoma constantly had a lower risk over time (0–19.5%). For patients with complete remission after initial treatment, prognosis varied by histological subtypes, with PTCL, NOS having a negative impact. Our data suggested that the risk stratification using the International Prognostic Index might not accurately predict the COS3 for survivors of PTCLs. The COS3 provided time-dependent prognostic information for PTCLs, representing a possible surrogate prognosis indicator for long-term survivors after systemic chemotherapy.

## INTRODUCTION

Peripheral T-cell lymphomas (PTCLs) are relatively rare and heterogeneous types of aggressive malignancies, accounting for only 10–30% of all cases of non-Hodgkin lymphomas (NHLs) [1–5]. Except for anaplastic lymphoma kinase (ALK)-positive anaplastic large-cell lymphoma (ALK+ALCL), the majority of subtypes have poor prognosis, with a 5-year overall survival (OS) of about 14–56% [6–9].

Previous studies of PTCLs made general predictions of prognosis based on clinical information at diagnosis [10, 11]. However, these estimates become less relevant as prognosis evolves. There are limited data for its prognostic value for patients surviving for a certain period of time. Conditional overall survival, a method providing more accurate information on survival outcome during follow-up, has been proposed [12, 13]. Generally, conditional survival estimates increase with the number of surviving years, especially for patients with advanced-stage disease [14]. These time-dependent statistics of conditional survival for patients over time since treatment can provide more accurate information during follow-up, and have been applied to lymphomas such as Hodgkin lymphoma [15], diffuse large B-cell lymphoma (DLBCL) [16], and NK/T cell Lymphoma (NK/TCL) [17].

However, there are limited data on the conditional survival for PTCLs. This retrospective study aimed to determine the spectrum of conditional survival and to estimate the annual hazards of death for patients with PTCLs.

## RESULTS

### Baseline characteristics

Baseline characteristics are summarized in Table 1. The median age was 45 years (interquartile range [IQR], 32–59 years), with a ratio of males to females of 2.3:1. NK/TCL was the most frequent subtype, accounting for 43.1%. In addition, 405 (62.3%) patients had advanced disease, and 303 (46.6%) patients were divided into the low-risk group according to the International Prognostic Index (IPI).

**Table 1:** Patient characteristics.

	**Total**	**AITL**	**PTCL, NOS**	**NK/TCL**	**ALK+ALCL**	**ALK-ALCL**	**Others**
Overall	650	91	65	280	55	33	126
**Sex**							
Male	455 (70.0%)	57 (62.6%)	49 (75.4%)	205 (73.2%)	41 (74.5%)	24 (72.7%)	82 (65.1%)
Female	195 (30.0 %)	34 (37.4%)	16 (24.6%)	75 (26.8%)	14 (25.5%)	9 (27.3%)	44 (34.9%)
**Age (yr)**							
< 60	502 (77.2%)	44 (48.4%)	49 (75.4%)	242 (86.4%)	52 (94.5%)	17 (51.5%)	98 (77.8%)
≥ 60	148 (22.8%)	47 (51.6%)	16 (24.6%)	38 (13.6%)	3 (5.5%)	16 (48.5%)	28 (22.2%)
**Stage**							
I-II	245 (37.7%)	1 (1.1%)	10 (15.4%)	163 (58.2%)	18 (32.7%)	20 (60.6%)	33 (26.2%)
III-IV	405 (62.3%)	90 (98.9%)	55 (84.6%)	117 (41.8%)	37 (67.3%)	13 (39.4%)	93 (73.8%)
LDH*							
< ULN	402 (61.8%)	38 (41.8%)	36 (55.4%)	194 (69.3%)	32 (58.2%)	22 (66.7%)	80 (63.5%)
≥ ULN	248 (38.2%)	53 (58.2%)	29 (44.6%)	86 (30.7%)	23 (41.8%)	11 (33.3%)	46 (36.5%)
**Extranodal sites**							
< 2	437 (67.2%)	73 (80.2%)	45 (69.2%)	161 (57.5%)	41 (74.5%)	26 (78.8%)	91 (72.2%)
≥ 2	213 (32.8%)	18 (19.8%)	20 (30.8%)	119 (42.5%)	14 (25.5%)	7 (21.2%)	35 (27.8%)
**ECOG**							
< 2	584 (89.8%)	74 (81.3%)	59 (90.8%)	257 (91.8%)	49 (89.1%)	32 (97/0%)	113 (89.7%)
≥ 2	66 (10.2%)	17 (18.7%)	6 (9.2%)	23 (8.2%)	6 (10.9%)	1 (3.0%)	13 (10.3%)
**Risk groups**							
Low	303 (46.6%)	19 (20.9%)	21 (32.3%)	154 (55%)	31 (56.4%)	19 (57.6%)	59 (46.8%)
Low-intermediate	192 (29.5%)	28 (30.8%)	23 (35.4%)	76 (27.1%)	16 (29.1%)	7 (21.2%)	42 (33.3%)
High-intermediate	111 (17.1%)	29 (31.9%)	17 (26.2%)	37 (13.2%)	5 (9.1%)	5 (15.2%)	18 (14.3%)
High	44 (6.8%)	15 (16.5%)	4 (6.2%)	13 (4.6%)	3 (5.5%)	2 (6.1%)	7 (5.6%)

AITL: angioimmunoblastic T-cell lymphoma; ALK: anaplastic lymphoma kinase positive; ALCL: anaplastic large cell lymphoma; PTCL: peripheral T-cell lymphoma; NKTCL: NK/T cell lymphoma. PTCL, NOS: peripheral T-cell lymphoma, unspecified. LDH: lactate dehydrogenase; ULN: upper limit of normal; ECOG: Eastern Cooperative Oncology Group.

*The upper limit of normal for LDH was defined as 240 IU/L.

Chemotherapy with an anthracycline-based regimen was administered to 528 (81.2%) patients, and radiotherapy was administered to 247 (38%) patients. The objective response rate (ORR) was 63.3%, with complete remission (CR) occurring in 42.8% (Table 2).

**Table 2:** First-line therapy regimens and response to initial therapy.

	**Total**	**AITL**	**PTCL, NOS**	**NK/TCL**	**ALK+ALCL**	**ALK-ALCL**	**Others**
Number	650	91	65	280	55	33	126
**First-line treatment**							
CHOP	211 (32.5%)	44 (48.4%)	18 (27.7%)	53 (18.9%)	19 (34.5%)	15 (45.5%)	62 (49.2%)
CHOP-EP/PEP	30 (4.6%)	0 (0%)	21 (32.3%)	1 (0.4%)	4 (7.3%)	2 (6.1%)	2 (1.6%)
CHOPE	99 (15.2%)	24 (26.4%)	14 (21.5%)	8 (2.9%)	29 (52.7%)	12 (36.4%)	12 (9.5%)
COP	12 (1.8%)	0 (0%)	0 (0%)	2 (0.7%)	0 (0%)	0 (0%)	10 (7.9%)
ASP-containing	217 (33.4%)	1 (1.1%)	2 (3.1%)	199 (71.1%)	0 (0%)	0 (0%)	15 (11.9%)
CHOP/GemOx	17 (2.6%)	11 (12.1%)	1 (1.5%)	0 (0%)	1 (1.8%)	1 (3.0%)	3 (2.4%)
CHOPE/GDP	13 (2.0%)	6 (6.6%)	2 (3.1%)	0 (0%)	0 (0%)	3 (9.1%)	2 (1.6%)
Other CT regimens	51 (7.8%)	5 (5.5%)	7 (10.8%)	17 (6.1%)	2 (3.6%)	0 (0%)	20 (15.9%)
Combined RT	247 (38%)	2 (2.2%)	14 (21.5%)	192 (68.6%)	3 (5.5%)	9 (27.3%)	27 (21.4%)
**Response to initial therapy**							
CR	278 (42.8%)	36 (39.6%)	18 (27.7%)	143 (51.1%)	36 (65.5%)	15 (45.5%)	30 (23.8%)
PR	133 (20.5%)	23 (25.2%)	14 (21.5%)	38 (13.6%)	7 (12.7%)	6 (18.2%)	45 (35.7%)
SD/PD	165 (25.4%)	28 (30.1%)	28 (43.1%)	57 (20.4%)	8 (14.5%)	7 (21.2%)	37 (29.4%)
Unknown	74 (11.1%)	4 (4.4%)	5 (7.7%)	42 (15%)	4 (7.3%)	5 (15.2%)	14 (11.1%)
**5-year OS**	50.0%	35.0%	28.0%	58.0%	82.0%	66.0%	40.0%

CHOP: cyclophosphamide doxorubicin vincristine prednisolone; CHOPE: CHOP plus etoposide; CHOP-EP/PEP: CHOP plus etoposide and cisplatin; COP: cyclophosphamide vincristine prednisolone; ASP-containing: COEPL (cyclophosphamide, vincristine, etoposide, prednisone and pegaspargase), CHOPL (CHOP plus pegaspargase), and LOP (pegaspargase, vincristine and prednisone); CHOPE/GemOx: CHOPE alternating with gemcitabine and oxaliplatin; CHOPE/GDP (CHOPE alternating with gemcitabine, cisplatin and dexamethasone); CT: chemotherapy; Other CT regimens: ESHAP (ESHAP: cytarabine, cisplatin, etoposide, methylprednisolone; COPP (cyclophosphamide vincristine procarbazine prednisolone); COMP (cyclophosphamide vincristine methotrexate prednisolone); CHEP (cyclophosphamide adriamycin etoposide prednisolone); Hyper CVAD/MA: cyclophosphamide, vincristine, doxorubicin, and dexamethasone alternating with methotrexate, and cytarabine); ITE (pirarubicin ifosfamide etoposide); FND (fludarabine mitoxantrone dexamethasone); GDP (gemcitabine dexamethasone cisplatin), SMILE (dexamethasone methotrexate ifosfamide l-asparaginase etoposide), and oral thalidomide. RT: radiation therapy; CR: complete response; PR: partial response; SD/PD: stable disease/progressive disease; AITL: angioimmunoblastic T cell lymphoma; ALK: anaplastic lymphoma kinase positive; ALCL: anaplastic large cell lymphoma; PTCL: peripheral T-cell lymphoma; NKTCL: NK/T cell lymphoma. PTCL, NOS: peripheral T-cell lymphoma, not otherwise specified.

### Overall survival, conditional survival, and annual hazard

**Figure 1:**
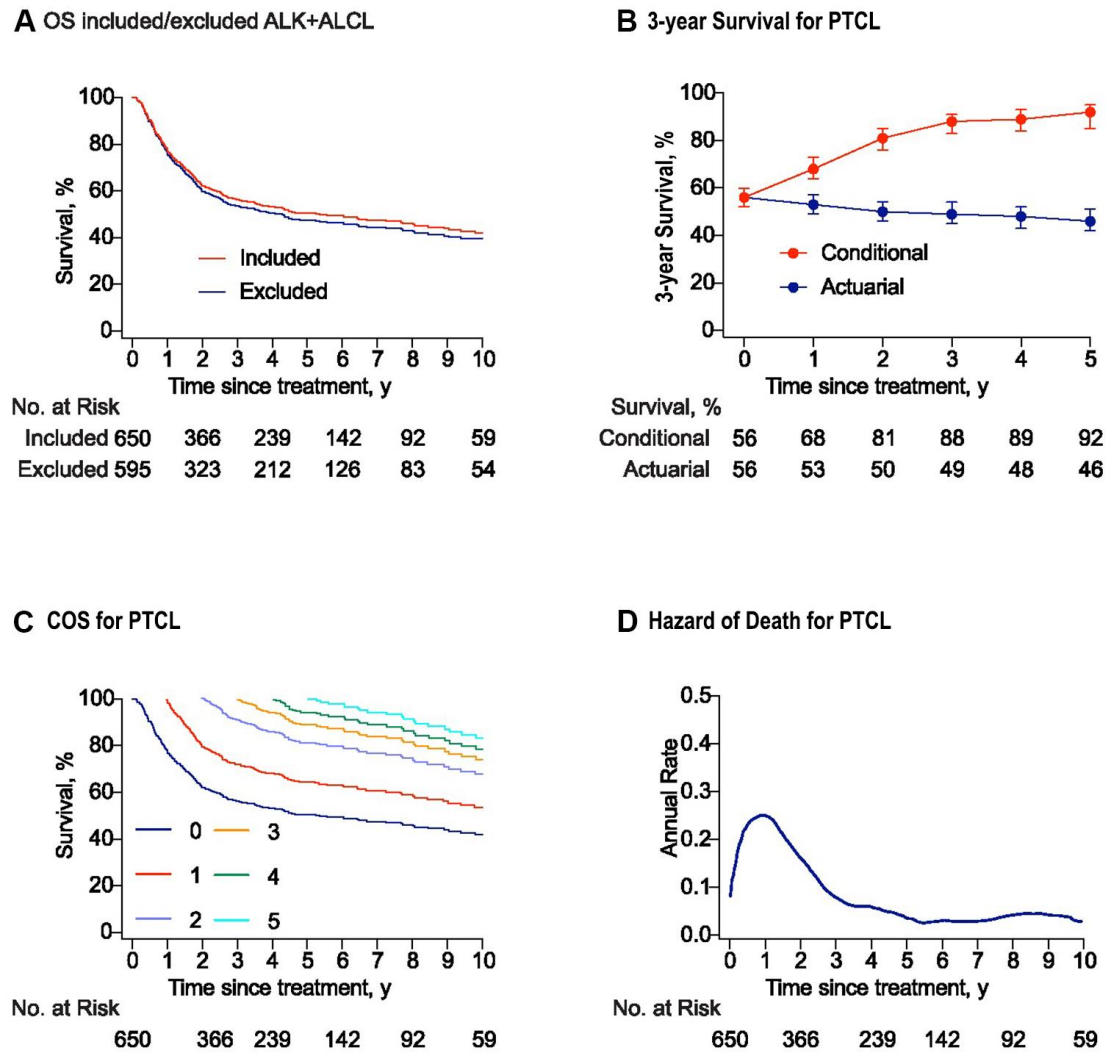
**Survival and conditional survival curves for patients with peripheral T-cell lymphomas (PTCLs).** (**A**) The overall survival curves for the whole study and the cohort with PTCL excluded anaplastic lymphoma kinase positive anaplastic large-cell lymphoma (ALK+ALCL). (**B**) Three-year conditional and 3-year actuarial survival with error bars of 95% confidence intervals (CIs) for the whole study cohort. For example, the 3-year actuarial survival rate at 2 years was the 5-year survival rate estimated at baseline. All the actuarial survival rates were calculated at the time point of starting treatment. (**C**) Conditional survival curves for patients who have survived for 1 year, 2 years, 3 years, 4 years, and 5 years from the time of treatment are shown. (**D**) Smoothed hazard plots for the annual rate of death for PTCLs since treatment.

With a median follow-up of 5.4 years, 320 (49.2%) patients died, and the majority of deaths (n = 235, 73.4%) occurred within the first two years after treatment. The 5-year OS rates were 50.3% (95% confidence interval [CI], 46.4–54.4%) for the whole cohort, and 47.5% (95%, CI: 45.3–49.7%) for those patients without ALK+ALCL, respectively (Figure 1A).

There-year conditional survival (COS3) was defined as the probability of surviving an additional 3 years for patients who had already survived for a certain time. For the whole cohort, the COS3 rates increased for the survivors, while actuarial OS decreased over time (Figure 1B). The conditional survival probabilities for patients with PTCLs increased obviously in the first two years after treatment and then increased slightly in the following years (Figure 1C). The COS3 at year 2 (the probability of reaching the landmark of year 5) increased to 81% (Δ 31% compared with 5-year OS). In addition, the overall annual death hazard of 24% in the first year was the highest, which then decreased to about 10% at year 2 (range: 0–24.2%). Moreover, the hazard after five years post- treatment showed a stable trend, with an annual death hazard of about 5% (Figure 1D).

### Conditional survival and annual hazards stratified by subtypes

**Figure 2:**
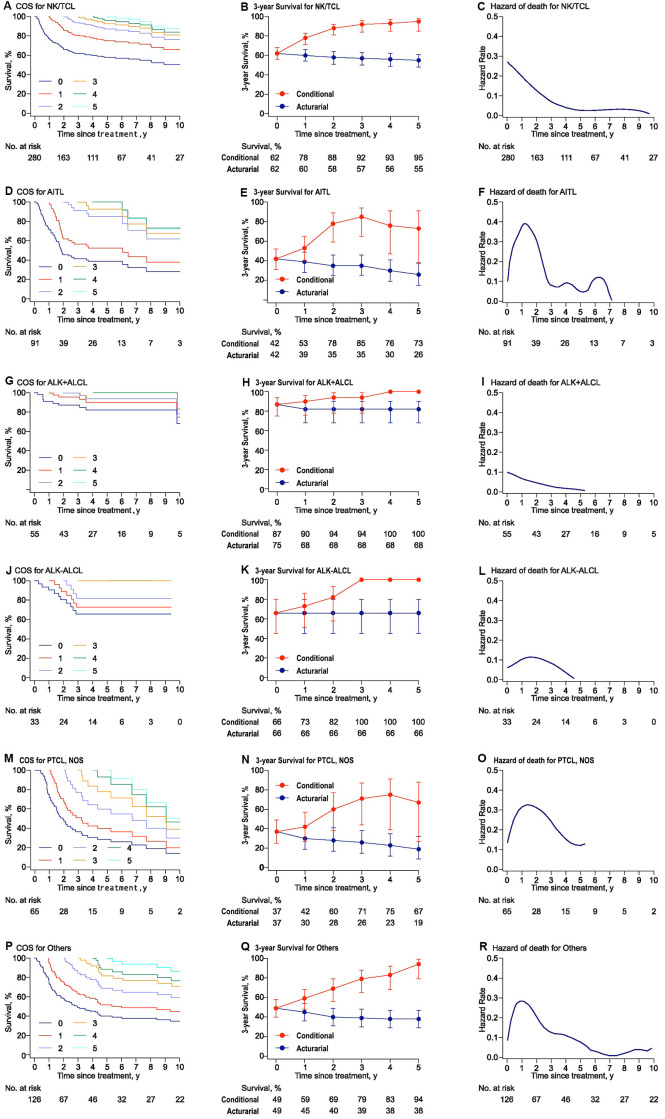
**Conditional survival, actuarial survival, and annual hazards stratified by histologic subtypes for PTCLs.** (**A**), (**D**), (**G**), (**J**), (**M**), and (**P**) presented the conditional survival curves for patients who have already survived a certain time for each subtype. (**B**), (**E**), (**H**), (**K**), (**N**), and (**Q**) showed the three-year conditional and 3-year actuarial survival with error bars of CIs. (**C**), (**F**), (**I**), (**L**), (**O**), and (**R**) demonstrated the smoothed hazard plots for the annual rate of death for each subtype. AITL: angioimmunoblastic T-cell lymphoma; ALK: anaplastic lymphoma kinase; ALCL: anaplastic large cell lymphoma; PTCL: peripheral T-cell lymphoma; NKTCL: NK/T cell lymphoma. PTCL, NOS: peripheral T-cell lymphoma, not otherwise specified.

Heterogeneous change in COS3 and the annual hazard of death varied among histological subtypes (Supplementary Figure 2). In general, COS estimates increased obviously over time in almost all subtypes. For 2-year survivors, the COS3 and annual hazard of death were 88% and < 7% for NK/TCL, 78% and < 10% for angioimmunoblastic T-cell lymphoma (AITL), 94% and <1% for ALK+ALCL, 82% and 20% for ALK-ALCL, 60% and 31% for peripheral T-cell Lymphoma, not otherwise specified (PTCL, NOS), and 69% and 16.5% for other histological types (Others), respectively (Figure 2).

### Conditional survival and annual hazards based on risk-stratification

**Figure 3:**
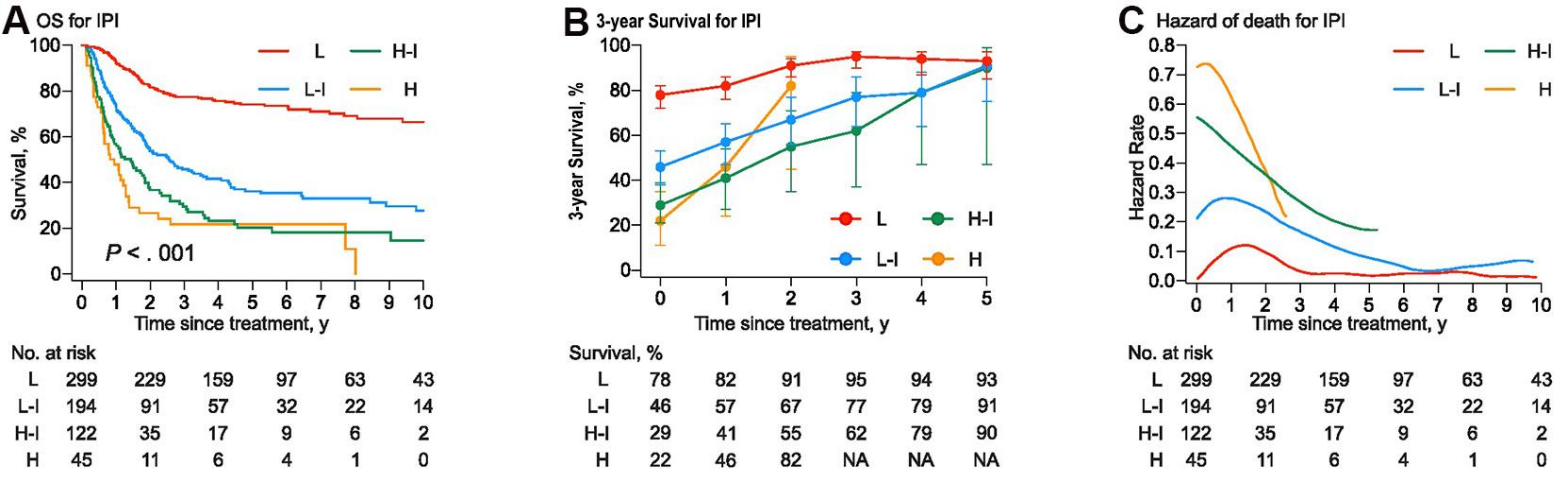
(**A**) The overall survival curves were stratified into four groups by the International Prognostic Index (IPI). (**B**) Three-year conditional survival with error bars of 95% CIs for patients who have survived for 1 year, 2years, 3 years, 4 years, and 5 years from the time of treatment. (**C**) Smoothed hazard plots for the annual rate of death since treatment.

The probability of survival increased more strikingly in higher-risk patients as time accrued (Figure 3). The COS3 and annual hazard of death at year 2 were 91% and 5.7% for the low-risk group, 67% and 16% for the low-intermediate risk group, 55% and 25% for the high-intermediate risk group, and 82% and 22% for high-risk group (Supplementary Table 1). For the high-intermediate patients who survived four years after inductive therapy, the COS3 was comparable with that of the low-intermediate risk group (79%). The 5-year survivors in PTCLs, except for the high-risk group, attained an equivalent favorable COS3 (all > 90% at year 5).

### Conditional survival and annual hazards of death for patients with CR

**Figure 4:**
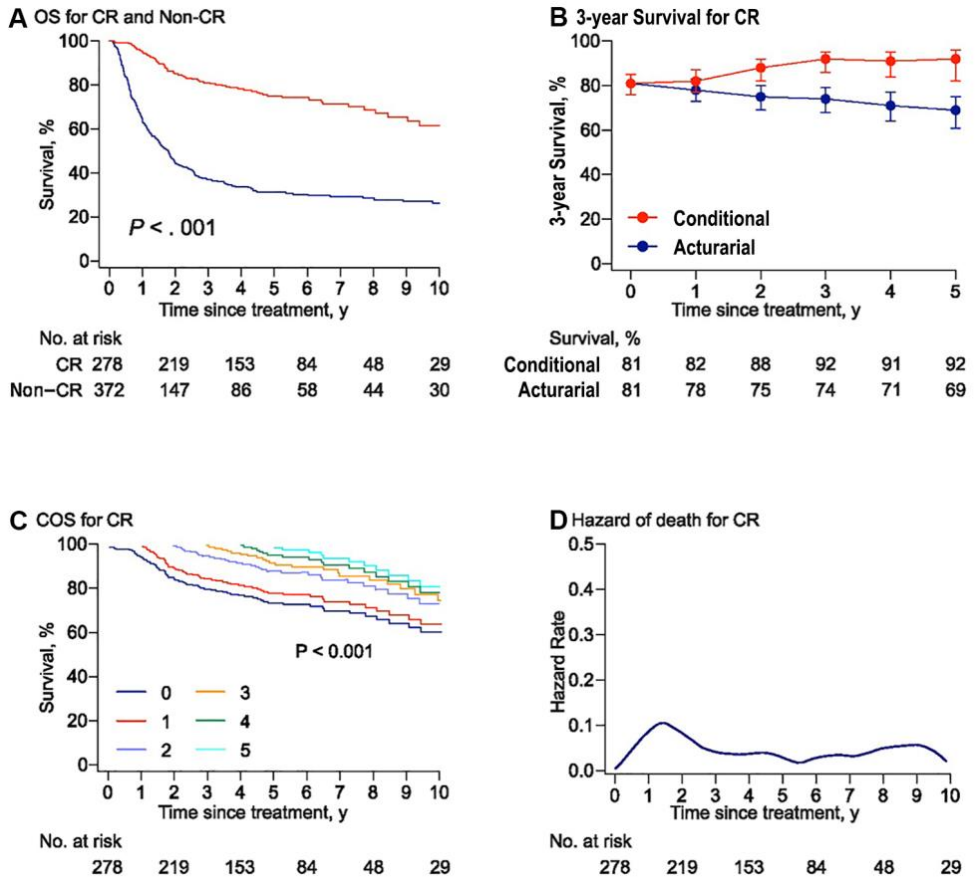
**Risk-dependent conditional survival and annual hazard for patients with CR.** (**A**) Overall survival curves for the patients who achieved or failed to obtain complete remission (CR) to initial chemotherapy. The overall survival rate was 75% and 31% at 5 years in the CR group and non-CR group, respectively. Survival comparison was made using the log-rank test. (**B**) Three-year conditional and 3-year actuarial survival with error bars of 95% confidence intervals (CIs) for the patients with CR. (**C**) Conditional survival curves for patients with CR who have survived for 1 year, 2 years, 3 years, 4 years, and 5 years from the time of treatment are shown. (**D**) Smoothed hazard plots for the annual rate of death for patients with CR since treatment.

Compared with those patients who could not achieve CR after initial treatment, patients who achieved CR had a better COS3 (Figure 4B, 4C). The annual hazard of death was present throughout and the highest risk of death was observed within the first year after treatment (about 13%) and was comparably stable at a low rate (< 5%) after year 3 (range: 0–13.5%, Figure 4D). For 2-year survivors, patients with PTCL, NOS had a worse prognosis, with a COS3 rate of 67% compared with those with ALK+ALCL (95%), NK/TCL (88%), and ALK-ALCL (85%, Supplementary Table 2).

## DISCUSSION

The disease burden of NHLs has been increasing in China over the last decade [18]. The present study presented the changes in survival probability based on a large-scale study of patients with PTCLs in the real world. The five-year OS of 50.3% in our study was favorable in comparison with other published studies [19, 20]. The main possible reason might be the large proportion of NK/TCL (43%) in our cohort. Specifically, the prognosis of early-stage NK/TCL has been improved by radiotherapy and chemotherapy regimens containing asparaginase in the past decade [21–23].

Our results revealed that the survival possibility increased dynamically with elapsed time after treatment for patients with PTCLs. COS3 increased markedly for most histological subtypes. Further analysis showed a histology-dependent pattern: AITL, PTCL, NOS, and NK/TCL had an initially higher hazard of death that decreased over time, whereas ALK+ALCL had a constantly lower risk. These findings represented a markedly heterogeneous spectrum of prognosis in PTCLs over time, providing time-dependent survival information for clinicians to make rational decisions on patients’ intervention selection and follow-up guidance.

The heterogenous prognosis of PTCLs varies by histological subtypes [7]. For example, patients with AITL were associated with poor prognosis, with a 5-year OS rate of less than 40% [3, 24, 25]. However, for AITL, the 3-year survival probability for two-year survivors reached 78% in our study. Furthermore, our results of a low annual hazard of death and a COS3 more than 90% for patients who still survived at 2 years after treatment, demonstrated a good long-term outcome in ALK+ALCL. Therefore, the second year after treatment could be a critical time point in the prognosis of patients with PTCLs, which might be associated with relapse [26].

Similar to the observations in previous research, a high IPI score and a lack of CR after systemic therapy resulted in inferior OS in PTCLs [27, 28]. Risk stratification could predict the initial prognosis of patients with PTCLs in this study, which was consistent with previous studies [29, 30]. Despite the heterogeneous prognoses in each risk category over time, the conditional survival is improved and the annual hazard of death decreased, more strikingly in the higher risk groups. At 5 years after treatment, the non-high-risk groups attained an equivalent and favorable COS3 (all > 90% at year 5). However, the results for COS3 in the high-risk group implied that risk stratification based on the IPI score might not be appropriate to predict conditional survival for patients with PTCLs. A possible explanation might be that the individual prognostic factors of IPI lost their predictive value when patients had already survived for a period of time [12]. Many patients with poor prognostic factors died in the first few years. Those who had initially unfavorable predictors but survived for a certain time after treatment were likely to achieve a better prognosis in the following years. Therefore, the results highlighted the importance of improving the current prognosis model for PTCLs. A more accurate prognostic scoring system, including genetic stratification, should be considered for survivors with PTCLs, as noted by previous studies [11, 31, 32]. Furthermore, the COS3 could be used as a surrogate end point to validate the accuracy of novel and advanced evaluation systems of risk stratification, from a dynamic perspective.

Our results further suggest that the patients who achieved CR after induction therapy and survived for more than two years might have an encouraging subsequent OS, similar to the results of a previous study [33]. A tendency for plateauing for the annual hazard of death in patients who achieved CR was observed after three years. However, our finding also suggests that the histological subtypes with initially inferior prognosis, such as PTCL, NOS, still retained a higher hazard of death in patients who achieved CR. As far as we know, durable remissions are uncommon with CHOP-based chemotherapy in some PTCLs [34]. A higher death risk in the previous two years might be associated with early relapse. Generally, our data provides information for the evaluation of therapeutic opportunities, such as new drugs or regimens, including allografts, at specific time points for patients who respond to intensive therapies, [35–38] and suggest that assessment of the COS3 might be used to stratify patients who achieved CR but remained at a high risk of early or late relapse.

There were several limitations of our study. First, the existence of heterogeneity of treatment patterns over a long-time span made it difficult to analyze the potential impacts of therapeutic approaches on outcomes of patients with PTCLs. Second, the central pathology of the histological subtypes could not be reviewed. Third, because of the small sample size in some histologic subtypes, the sample size should be further expanded to confirm the results. In conclusion, PTCL is a heterogeneous disease with multiple subtypes and varying clinical outcomes among patients who survive after a certain time. Multidisciplinary collaboration is necessary sometimes for better outcome, as rarer conditions in other disease [39, 40].

However, an apparent additional three-year survival improvement was observed in most subtypes after two years, which was associated with a reduced annual hazard of death. Accordingly, the follow-up interval might be varied among different histological subtypes of PTCLs based on our data. In addition, a significant difference was observed when evaluating the prognosis of patients with PTCLs using the traditional OS method and the COS3 method. Therefore, these results indicated that a conditional survival strategy and annual hazard analyses might be more accurate and helpful during additional survival years, especially for patients with initially inferior histological subtypes. Generally, the results suggest that COS3 might be useful in clinical decision making, novel prognosis model assessment, biomarker validation, patient counseling, disease surveillance, and clinical trial improvement in patients with PTCLs.

## MATERIALS AND METHODS

### Patients

The study was approved by the ethics committee of Peking University Cancer Hospital and Institute, and an exemption for individual informed consent from the patients was granted because of the anonymous nature of the data.

A total of 679 patients newly diagnosed with PTCLs at Peking University Cancer Hospital and Institute who were treated with combination chemotherapy from January 1997 to January 2016 were reviewed. Twenty-nine cases were excluded because of ambiguous histological type (n = 13) and incomplete clinical data (n = 16). Finally, 650 cases were enrolled (Supplementary Figure 1). The disease stage was determined using the Ann-Arbor staging system, and risk stratification was performed on the International Prognostic Index (IPI) [41]. Response to the treatment was reported accordingly [42, 43].

OS was defined as the time from treatment to death from any cause or the last follow-up. Conditional overall survival was defined as the probability of surviving an additional number of years, given that the patient had already survived a certain number of years since primary treatment [14, 44]. For example, the 2-year COS3 was defined as the probability of surviving an additional 3 years for a patient who had already survived 2 years (surviving to the landmark of 5 years since treatment).

### Statistical analysis

Median follow-up time was estimated on OS using the reverse Kaplan–Meier method. The survival probability was estimated using the Kaplan–Meier method, and survival differences between groups were tested using the log-rank test for statistical significance. The annual hazard of death was defined as the rate of death during a certain year after treatment for surviving patients. Smoothed hazard estimates were calculated based on the Kernel–Epanechnikov smoothing procedure [45].

All statistical tests were two-sided, with an alpha level of 0.05 as the significance cutoff. All analyses were conducted using IBM SPSS Statistics 25.0 (IBM Corp., Armonk, NY, USA) and R version 3.2.5 (https://www.r-project.org).

## Supplementary Material

Supplementary Figures

Supplementary Tables
