# Supplementary material for: Conditional survival and hazards of death for peripheral T-cell lymphomas
Source: Aging (Albany NY). 2021 Mar 26;13(7):10225–39. doi: 10.18632/aging.202782 (PMC8064157; doi:10.18632/aging.202782)
Supplement: Supplementary Figures [file aging-13-202782-s001.pdf]

SUPPLEMENTARY FIGURES

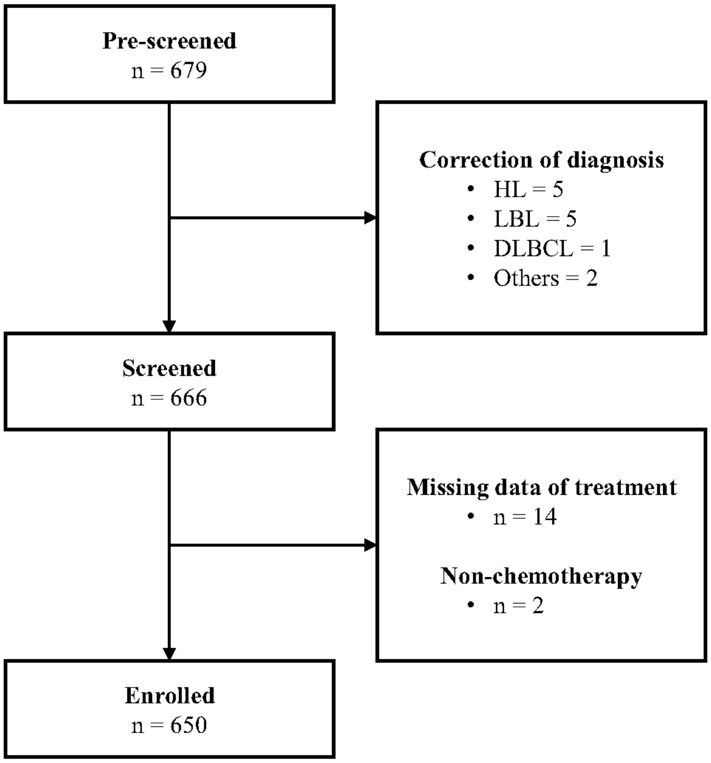

Supplementary Figure 1. Flow chart for screening eligible patients.

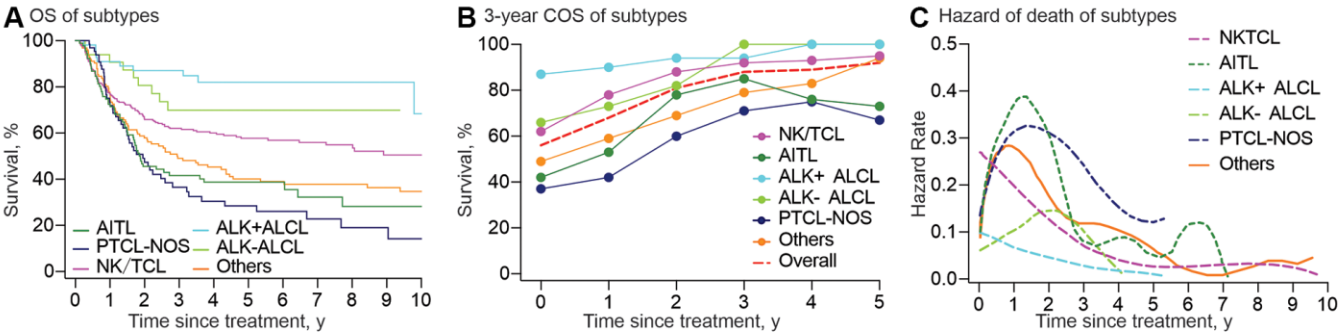

Supplementary Figure 2. Summary and comparison for different histologic subtypes of PTCLs. (**A**) Overall survival for each histologic subtype. (**B**) Three-year conditional survival for each histologic subtype. The red line represented the 3-year conditional survival for the whole cohort. (**C**) Smoothed hazard plots for the annual rate of death for each histologic subtype.
