# Supplementary material for: Conditional survival and hazards of death for peripheral T-cell lymphomas
Source: Aging (Albany NY). 2021 Mar 26;13(7):10225–39. doi: 10.18632/aging.202782 (PMC8064157; doi:10.18632/aging.202782)
Supplement: Supplementary Tables [file aging-13-202782-s002.pdf]

SUPPLEMENTARY TABLES

Supplementary Table 1. The three-year conditional overall survival probabilities for different risk groups over time.

	Time since treatment				
	**1 year**	**2 year**	**3 year**	**4 year**	**5 year**
**Risk groups**					
**Low-risk**					
3-year COS (%)	82	91	95	94	93
95%CI (%)	76-86	86-94	90-97	87-97	85-97
**Low-intermediate-risk**					
3-year COS (%)	57	67	77	79	91
95%CI (%)	47-65	56-77	64-86	64-88	75-97
**High-intermediate-risk**					
3-year COS (%)	41	55	62	79	90
95%CI (%)	27-39	35-54	37-71	47-79	47-93
**High-risk**					
3-year COS (%)	46	82	NA	NA	NA
95%CI (%)	24-65	45-95	NA	NA	NA

Supplementary Table 2. Three-year conditional overall survival probabilities for each histological subtype in PTCLs and the patients with CR over time.

	Time since treatment				
	1 year	2 year	3 year	4 year	5 year
**All patients**					
3-year COS (%)	68	81	88	89	92
95%CI (%)	64-73	76-85	83-91	84-93	85-95
**Patients with CR**					
3-year COS (%)	80	87	91	90	91
95%CI (%)	74-85	80-91	85-95	81-95	80-96
**NK/TCL**					
3-year COS (%)	78	88	92	93	95
95%CI (%)	71-83	81-92	84-96	85-97	85-98
**Patients with CR**					
3-year COS (%)	84	88	92	93	94
95%CI (%)	76-90	79-93	84-97	82-97	78-98
**AITL**					
3-year COS (%)	53	78	85	76	73
95%CI (%)	40-65	59-89	65-94	47-91	37-91
**Patients with CR**					
3-year COS (%)	74	94	94	73	62
95%CI (%)	56-86	67-99	67-99	36-91	21-86
**ALK-ALCL**					
3-year COS (%)	73	82	100	100	100
95%CI (%)	51-86	58-93	100-100	100-100	100-100
**Patients with CR**					
3-year COS (%)	79	85	NA	NA	NA
95%CI (%)	47-93	51-96	NA	NA	NA
**ALK+ALCL**					
3-year COS (%)	90	94	94	100	100
95%CI (%)	76-96	78-99	78-99	100-100	100-100
**Patients with CR**					
3-year COS (%)	95	95	95	100	100
95%CI (%)	71-99	71-99	71-99	100-100	100-100
**PTCL-NOS**					
3-year COS (%)	42	60	71	75	67
95%CI (%)	27-57	38-77	44-87	39-91	27-88
**Patients with CR**					
3-year COS (%)	58	67	61	74	83
95%CI (%)	30-78	34-86	25-83	29-93	27-97
**Others**					
3-year COS (%)	59	69	79	83	94
95%CI (%)	48-68	56-79	65-88	68-92	79-99
**Patients with CR**					
3-year COS (%)	84	83	93	93	NA
95%CI (%)	59-95	55-94	59-99	59-99	NA

AITL: angioimmunoblastic T-cell lymphoma; ALK: anaplastic lymphoma kinase positive; ALCL: anaplastic large cell lymphoma; PTCL: peripheral T-cell lymphoma; NKTCL: NK/T cell lymphoma. PTCL, NOS: peripheral T-cell lymphoma, unspecified. LDH: lactate dehydrogenase; ULN: upper limit of normal. CR: complete response.
